# A Comparative Study of the Gas Sensing Behavior in P3HT- and PBTTT-Based OTFTs: The Influence of Film Morphology and Contact Electrode Position

**DOI:** 10.3390/s140916869

**Published:** 2014-09-11

**Authors:** Kyriaki Manoli, Liviu Mihai Dumitru, Mohammad Yusuf Mulla, Maria Magliulo, Cinzia Di Franco, Maria Vittoria Santacroce, Gaetano Scamarcio, Luisa Torsi

**Affiliations:** 1 Dipartimento di Chimica Università degli Studi di Bari Aldo Moro, Via Orabona 4, 70126 Bari, Italy; E-Mails: kyriaki.manoli@uniba.it (K.M.); liviu.dumitru@uniba.it (L.M.D.); yusuf.mulla@uniba.it (M.Y.M.); maria.magliulo@uniba.it (M.M.); 2 CNR-IFN and Dipartimento Interateneo di Fisica, Università degli Studi di Bari “A. Moro”-Via Orabona 4, 70126 Bari, Italy; E-Mails: cinzia.difranco@uniba.it (C.D.F.); maria.santacroce1@uniba.it (M.V.S.); gaetano.scamarcio@uniba.it (G.S.)

**Keywords:** organic thin film transistors, chemical sensors, volatile organic compounds

## Abstract

Bottom- and top-contact organic thin film transistors (OTFTs) were fabricated, using poly(3-hexylthiophene-2,5-diyl) (P3HT) and poly[2,5-bis(3-tetradecylthiophen-2-yl)thieno[3,2-b]thiophene] (PBTTT-C16) as *p*-type channel semiconductors. Four different types of OTFTs were fabricated and investigated as gas sensors against three volatile organic compounds, with different associated dipole moments. The OTFT-based sensor responses were evaluated with static and transient current measurements. A comparison between the different architectures and the relative organic semiconductor was made.

## 1. Introduction

Organic thin film transistors (OTFTs) have attracted increasing attention due to their wide variety of potential sensing applications, such as environmental monitoring [1–4], food industry and medical diagnostics [5,6]. Multiple parameters, such as mobility, threshold voltage and bulk conductivity, can be used for recognition/sensing of analyte molecules [7]. A transistor-based sensor is above all an amplifier, thus enabling the development of sensing devices with high sensitivity and a low detection limit [8,9]. In addition, OTFTs can be fabricated on flexible or transparent substrates by different methods suitable for low-cost and mass production [10]. Their versatile design along with the possibility to tune the chemical and physical properties of the materials involved can improve further their sensitivity and selectivity [6,11].

Multiple configurations exist, and each of them is determined by the specific application. Among them, the bottom-gate (BG) structure is considered as the most effective architecture of OTFTs for gas sensing applications. In this case, the semiconductor is both the active and sensing layer. The source-drain contacts can either be deposited on top of the semiconducting layer, usually referred to as the top-contact bottom-gate (TCBG) configuration, or formed on the dielectric, prior to the deposition of the semiconductor, known as the bottom-contact bottom-gate (BCBG) configuration. Charge injection at metal-organic semiconductor interfaces depends on the energy level adjustment between the two materials. The positioning of the charge injecting source-drain contacts with respect to the charge accumulation layer at the interface of the organic semiconductor and the dielectric plays a significant role in the electrical performance of the device. In terms of electrical performance, the BCBG structure is believed to have a higher contact resistance than TCBG [12,13]. Such an effect is generally attributed to the morphological discontinuity of the semiconductor in BCBG compared to the TCBG configuration. Moreover, upon deposition of the metal contact on top of the semiconductor, penetration of metal clusters into the semiconductive layer can occur, thus enabling the reduction of contact resistance [14].

In general, the architecture of the OTFT device plays a critical role in sensing applications. However, few reported data can be found on how the arrangement of the metal contacts influences the performance of transistor-based sensors [15]. Herein, the bottom- and top-contact structures of OTFTs are investigated as sensors in the presence of the vapors of volatile organic compounds (VOCs). More specifically, we have investigated how the different architectures (TCBG and BCBG) can influence the sensitivity of the OTFT-based sensors, when exposed to the vapors of n-butanol, ethanol and acetone. Two p-type organic semiconductors (OSs) are studied. Furthermore, a morphological characterization of the obtained films is made, providing useful information that helps to understand better the differences in terms of electrical and sensing performance. To the best of our knowledge, the effect of the metal contacts placement with respect to the semiconducting layer on the sensing performance upon exposure to VOCs has not been previously investigated. A comparison of the response to selected VOCs between poly(3-hexylthiophene-2,5-diyl) (P3HT) and poly[2,5-bis(3-tetradecylthiophen-2-yl)thieno[3,2-b]thiophene] (PBTTT-C16) is made for the first time, as well.

## 2. Experimental Section

**Figure 1.**
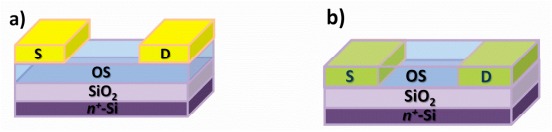
Schematic illustration of (**a**) top-contact bottom-gate (TCBG) (**b**) bottom-contact bottom-gate (BCBG) organic thin film transistors (OTFTs).

OTFT fabrication: TCBG and BCBG OTFTs were fabricated. All structures investigated in the present study were fabricated on highly n-doped silicon that serves as the gate electrode. Thermally grown silicon dioxide of 300 nm thickness serves as the gate dielectric. The substrates were rinsed with water/acetone/water, then dipped in an isopropanol ultrasonic bath for 15 min and dried under nitrogen (N_2_) flow. Two commercially available p-type organic semiconductors were used as active layers: P3HT, regioregular, purchased from Rieke Metals, was dissolved in chloroform (2.5 mg/mL). PBTTT-C16, electronic grade, purchased from Ossila Ltd, was dissolved in a mixture of 1,2-dichlorobenzene:chloroform (9:1). In this case, the concentration of PBTTT-C16 was 3 mg/mL. Both organic semiconductors were deposited via spin coating at 2000 and 3000 rpm, respectively, for 60 s, forming a layer of a few nanometers (∼20 nm). After the deposition of PBTTT-C16, the samples were annealed at 150 °C for 10 min. The TCBG OTFT structure is illustrated in Figure 1a. Gold contacts of 100 nm thickness were thermally evaporated on top of each semiconductor, through a shadow mask of parallel electrodes. The length (L) and width (W) of the channel is 200 μm and 4 mm, respectively. For the BCBG structure (Figure 1b), gold parallel electrodes were deposited on SiO_2_, using e-beam evaporation. In this case, 5 nm of titanium were deposited as the promoting layer to improve the adhesion of gold on the SiO_2_. The thickness of the gold contacts was 50 nm. The resulting BCBG TFTs were of the same channel length and width as the TCBG TFTs. The electrodes dimensions match those of the transistor's channel. The devices were fabricated and measured in a standard laboratory environment and operated at room temperature.

OTFT measurements: Electrical measurements were carried out using the Keithley Model 4200-SCS semiconductor parameter analyzer. Initially, the OTFTs were characterized by measuring the I_DS_-V_DS_ output characteristics, where the drain to source current (I_DS_) is recorded at different gate voltages (V_G_) ranging from +20 to −100 V with a step of −20 V, while the source and drain bias (V_DS_) is swept from 0 to −100 V. The electric properties of the OTFTs were extracted from the obtained output curves using the conventional field effect transistor (FET) equation (Equation (1)) in the saturation regime.

(1) $$I_{DS}^{sat.}=\frac{W}{2L}C_{i}\mu {\left(V_{G}-V_{T}\right)}^{2}$$

where W and L are the width and length of the channel, C_i_ = 9 nF/cm^2^ is the capacitance of the dielectric, μ is the mobility and V_T_ is the threshold voltage.

**Figure 2.**
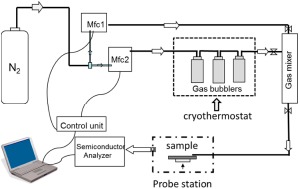
Schematic illustration of the gas sensing apparatus.

Analyte vapor generation: For the vapor sensing measurements, controlled concentrations of analyte vapors were generated using a gas delivering unit, as shown in Figure 2. The gas delivery unit involves a dry nitrogen flux that splits into two parts. The first line carries pure N_2_, and the second line passes N_2_ through three sealed glass bubblers in series that contain the analyte in its liquid form. Each flow is controlled by two mass flow controllers (Brooks Smart DMFC model 5850C). The bubblers are immersed in a cryothermostat kept at –6.6 °C. The desired analyte concentration, expressed from now on as activity a_S_ (a_s_ = p/p_sat_, where p is the pressure of the vapors and p_sat_ is the saturation vapor pressure at −6.6 °C), is generated by mixing nitrogen with saturated vapors of the analyte at atmospheric pressure. The measurements were performed at relatively high analyte concentrations, since this condition allowed for better discrimination of the effects occurring. The analyte we have employed in this study has different saturated vapor pressures. Due to the different volatility of each compound, under the same pressure and temperature, the value of activity corresponds to different amounts of ppm_V_. In this case, a_s_ = 0.5 is equal to 391, 5725 and 31,960 ppm_V_ for *n*-butanol, ethanol and acetone, respectively. The mixture passes at a rate of 200 mL/min through a nozzle directly onto the surface of the active layer.

Herein, we studied the response of each sensor subjected to the same activity (a_s_ = 0.5) of *n*-butanol, ethanol and acetone vapors. Ethanol and *n*-butanol are two primary alcohols bearing a similar dipole moment (*i.e*., p_n-butanol_ = 1.66D and p_ethanol_ = 1.69D), but differ in the length of the alkyl chain; meanwhile, acetone is selected due to the higher dipole moment (p_acetone_ = 2.91D). All of the reagents, solvents included, were purchased from Aldrich and used without further purification.

Vapor sensing measurements: The response of each OTFT sensor was measured by carrying out static and transient current measurements. In each measurement, pulses of alternating polarity were applied to the gate of the transistor, while its drain was biased at a certain voltage. This technique is described in detail in [16]. For the static I_DS_-V_G_ transfer characteristics, the pulse amplitude was gradually increased from 0 to 100 V with a step of 10 V, while alternating the polarity at each step. The V_DS_ was kept constant at −80 V. The pulse duration t_on_ was 10 ms, and the time that the device was not biased is t_off_ = 0.1 s. Pulsed transient current measurements were also realized by biasing the device with a negative gate voltage V_G_ = −100 V, followed by a positive voltage of +20 V (V_DS_ = −80 V). Each pulse lasted 10 ms, while among the pulses, the device remained unbiased for 2 s.

The saturation drain current was recorded in nitrogen and in a controlled analyte concentration. Static measurements were realized by recording the I_DS_-V_DS_ transfer characteristics prior (under N_2_ flow) and while exposing the devices to the analyte's vapors. The vapor exposure time was 10 min, which was enough for the sensor to reach the equilibrium state. The recovery of the signal was recorded after passing N_2_ at the same time. For the transient drain measurements, drain current changes upon three successive equilibration at the same vapor activity (a_s_ = 0.5) with intervening recovery steps back to a_S_ = 0, by passing pure N_2_, were monitored over time.

The relative response of the maximum drain current at saturation for all sensors was calculated based on the following type (Equation (2)):

(2) $$\frac{\Delta I_{\dot{D}S}}{I_{DS_{0}}}=\frac{I_{DS_{0}}-I_{DS_{analyte}}}{I_{DS_{0}}}$$

where I_DS0_ and I_DSanalyte_ are the maximum saturation drain current under N_2_ flow (baseline) and after analyte exposure, respectively.

Scanning electron microscopy (SEM) characterization: The morphological characterization of PBTTT-C16 and P3HT was performed by a ∑igma Zeiss field emission scanning electron microscope (FESEM). The probing e-beam was set with an acceleration voltage of 12 kV, a 10-μm aperture size and a 1.5-mm working distance. SEM images were acquired by tilting the samples at a 65° angle and using the in-lens (secondary electrons) detector. The contrast of SEM images was adjusted using ImageJ 1.42R (National Institutes of Health USA) software.

## 3. Results and Discussion

### 3.1. OTFTs Electrical Performance

**Figure 3.**
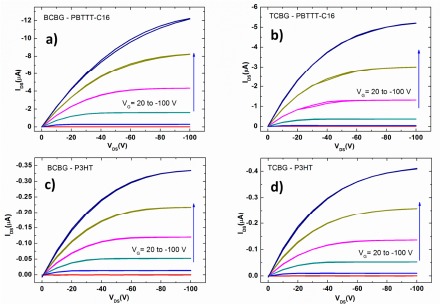
Double current-voltage I_DS_-V_DS_ output characteristics under nitrogen flux of: using poly[2,5-bis(3-tetradecylthiophen-2-yl)thieno[3,2-b]thiophene] (PBTTT-C16) (**a**), BCBG (**b**), TCBG; and poly(3-hexylthiophene-2,5-diyl) (P3HT) (**c**) BCBG, (**d**) TCBG. Gate voltages go from +20 V to −100 V in steps of −20 V.

Representative output I-V characteristics of the TCBG and BCBG OTFTs for the two organic semiconductors are depicted in Figure 3. A fairly good gate modulation is observed in all devices, with negligible hysteresis between forward and reverse scans. The average values of the figures of merit for all OTFT devices are summarized in Table 1. Mobility μ and threshold voltage V_T_ were graphically estimated in the saturation regime from the linear plot of the square root of the absolute I_DS_ *versus* V_G_, taking the slope and intercept, respectively.

Similar figures of merits were found by comparing TCBG and BCBG OTFTs. As for the two different semiconductors, the PBTTT-C16-based devices exhibited higher mobility than P3HT, while the average current amplification reached 10^4^ for PBTTT-C16 and 10^3^ for P3HT. In general, PBTTT-C16 is more stable and has higher mobilities relative to P3HT. Carrier mobilities up to 1 and 0.1 cm^2^/Vs have been previously reported for PBTTT and P3HT, respectively [17,18].

**Figure 4.**
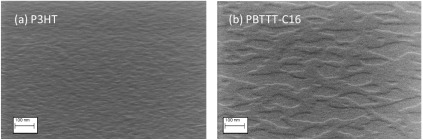
Topographical scanning electron microscope view of the two semiconductors: (**a**) P3HT and (**b**) PBTTT-C16. The probing e-beam was set with an acceleration voltage of 12 kV, a 10-μm aperture size and a 1.5-mm working distance. SEM images were acquired by tilting the samples at a 65° angle and using the in-lens (secondary electrons) detector.

The improved performance has been attributed mainly to their different morphological structure [17]. The surface morphology of PBTTT-C16 and P3HT films was investigated by means of scanning electron microscopy (SEM) analysis. The SEM images of each film on the Si/SiO_2_ are shown in Figure 4. The film of PBTTT-C16 consists of larger crystalline domains compared to P3HT. These large crystalline domains extend over several hundreds of nanometers. The film of P3HT presents more dense and small in size crystalline domains, with a nodular-like structure. It has been shown that by thermal annealing the PBTTT-C16 films to their liquid-crystalline state and then gradually cooling, larger crystalline domains are obtained. Liquid crystalline polymers have the potential of transforming a less ordered nodular film to one with increased crystalline domain sizes, which leads to improved transistor performance [18,19].

### 3.2. Vapor Sensing Measurements

**Figure 5.**
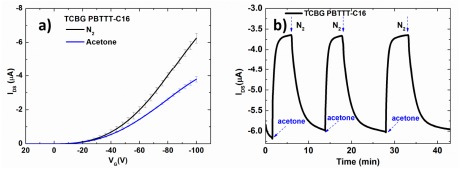
(**a**) Current-voltage I_DS_-V_G_ transfer characteristics upon nitrogen and after acetone (a_s_ = 0.5 at −6.6 °C) exposure of TCBG PBTTT-C16. Each transfer curve corresponds to average current values of three exposures. (**b**) Transient drain current (I_DS_) changes upon three successive equilibration at acetone vapors (a_s_ = 0.5 at −6.6 °C) with intervening recovery steps by passing pure N_2._

All devices were exposed to saturated vapors of acetone, ethanol and *n*-butanol of the same activity (α_s_ = 0.5). As shown in the examples of Figure 5, the sensor exhibits a fast response time and high repeatability. Moreover, a full recovery of the signal to its baseline is obtained when the device is subjected to N_2_ after each exposure, since reversible, weak interactions are involved in the sensing process. A decrease of the drain current is observed upon exposure of p-type-based OTFT to polar analyte vapors. Polar molecules can be entrapped at the grain boundaries and interact with charge carriers (holes), reducing the hole mobility [20]. The sensing mechanism is generally attributed to the potential barrier increase at grain boundaries upon exposure to the analyte and/or by a charge trapping effect. The analyte molecules can be physically adsorbed on the surface of the sensitive/semiconductive layer and/or percolate through the voids around the grains till they reach the dielectric surface [3,21]. The degree of physisorption depends on the chemical affinity between the active layer and the analyte, and it occurs both in the bulk and at the interfaces [9].

#### 3.2.1. Comparison of P3HT and PBTTT-C16 Vapor Sensing Performance

**Figure 6.**
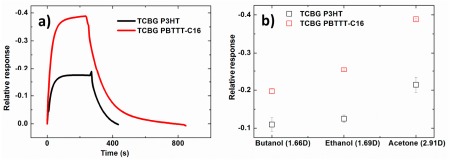
(**a**) Dynamic relative response of TCBG PBTTT-C16- (red line) and P3HT- (black line) based sensors subjected to acetone vapors (α = 0.5 at −6.6 °C). (**b**) Relative response in equilibrium of TCBG PBTTT-C16- (red open squares) and P3HT- (black open squares) based sensors *versus* all three analytes. The relative responses are determined from the transient current measurements. Error bars represent the standard error of mean experimental values, derived from three measurements.

The dynamic relative response of the drain current of the two semiconductors bearing a TCBG configuration upon exposure to acetone atmosphere is shown in Figure 6a. The response of the PBTTT-C16 sensors is higher compared to P3HT. This was true for all analytes, as can be seen in Figure 6b, where the relative response in equilibrium of the drain current of PBTTT-C16 and P3HT-based sensors towards the three analytes is presented.

The higher sensitivity of PBTTT-C16-based devices suggests that the analyte molecules interact more strongly with PBTTT-C16. This difference may be related to the chemical nature of the organic semiconductor and to the differences in the morphological characteristics of the two films. We saw that PBTTT-C16 film has a more three-dimensional structure, due to the formation of large crystalline domains. Consequently, a higher amount of analyte molecules can be adsorbed between the grains or diffuse through the larger voids down to the semiconductor/dielectric interface.

The response of both PBTTT-C16 and P3HT is higher towards acetone and lower in the presence of n-butanol vapors. The current response increases as we move to analytes with a higher dipole moment. Such an observation suggests that the sensing mechanism in these two systems have a common origin and can be probably associated with the dipolar interaction of analytes with the sensing layer [22]. It has been previously shown that the analyte molecules of stronger dipole moments can produce stronger responses from the same OTFT chemical sensor, since the charge transport is sensitive to the local polar environment [23].

#### 3.2.2. Comparison of Vapor Sensing Performance between BCBG and TCBG OTFTs

**Figure 7.**
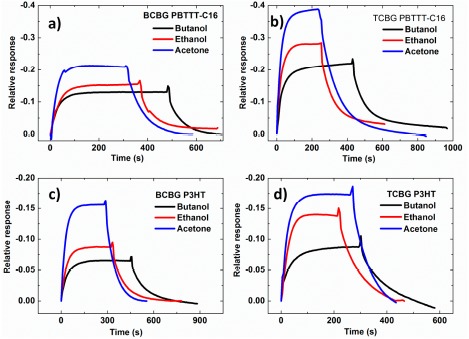
Dynamic relative response of the drain current *vs.* time of the OTFT-based sensors subjected to the three analytes at the same activity (a_s_ = 0.5 at −6.6 °C). (**a**) TCBG PBTTT-C16. (**b**) BCBG PBTTT-C16. (**c**) TCBG P3HT. (**d**) BCBG P3HT.

**Figure 8.**
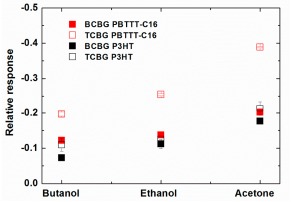
Relative response in equilibrium of all OTFTs-based sensors *versus* the three analytes: BCBG PBTTT-C16 (red full squares), TCBG PBTTT-C16 (red open squares), BCBG P3HT (black full squares) and TCBG P3HT (black open squares). The responses are evaluated based on the transient drain measurements. Error bars represent the standard error of mean experimental values, derived from three measurements.

In Figure 7, the dynamic relative response of the drain current for PBTTT-C16 and P3HT OTFTs bearing BCBG and TCBG configurations in all analytes is reported. Interestingly, an enhancement of the sensors' response is observed in the case of TCBG structures for both semiconductors. This is more evident for the PBTTT-C16-based OTFTs, where the response is almost doubled for all analytes, as seen in Figure 8, in the plot of the relative responses of drain current in equilibrium for all analyte-OS systems and types of structures.

Generally, the OTFT-based sensor response is mainly attributed to changes in the 2D conductance within the channel area. Additionally, in the case of the top-contact configuration, the response can be also influenced by the reduction in the bulk conductivity of the OS due to the trapping of analyte molecules between grain boundaries. As already mentioned, source-drain contacts in TC structures are not in the same plane with the 2D channel formed at the interface of the organic semiconductor and dielectric. Consequently, the carriers injected from the metal to the organic semiconductor have to cross through the bulk of the OS to reach the channel. Hence, the bulk conductivity changes contribute to the decrease of the drain current.

Another possible reason for the observed enhancement in the response of TC structures could be the adsorption of the polar analyte molecules on the bare gold electrode surface, possibly altering the work function (φ_f_). In the BCBG structure, the metal contacts are covered by the semiconductor layer, which acts as a barrier layer inhibiting the direct interaction of the analyte with the gold surface. It has been reported that vapor polar analytes (e.g., water [24], ammonia [25,26]) can be physically adsorbed on the bare surface of gold electrodes, resulting in a drop of the metal work function [27]. Variations in the φ_f_ of the metal contact due to the adsorption of analyte molecules can affect the charge injection of carriers between the contact and organic semiconductor. As mentioned earlier, the dimensions of the electrodes and the channel are the same (*i.e*., 200 μm × 4 mm) in this study. The large surface of the metal contacts can adsorb a higher amount of analyte molecules compared to devices where the electrodes dimensions are much smaller that the channel. Such effects can contribute to the overall decrease in the conductance of the device, giving rise to the chemical sensing effect.

A higher response in the presence of moisture in the bottom contact structure of pentacene-based transistors has been reported by D. Li *et al.* [15]. The observed behavior was attributed to the increased number of grain boundaries occurring in the coplanar structure near the contacts and to the higher contact resistance due to the moisture residing at the metal/OS interface, either setting apart the semiconductor from the contact or inducing a surface dipolar barrier. However, this was not the case for longer channel length devices (L = 200 μm), because the overall number of grain boundaries between the source and drain was similar in the two cases. It is well established that the vapor sensing behavior of short channel length organic transistors is different from longer ones, especially when the size of the grains is comparable or larger to that of the channel length [22,28].

## 4. Conclusions

In summary, we investigated the response of TCBG and BCBG OTFT-based sensors to polar analyte vapors, fabricated with different p-type organic semiconductors. In all cases, a decrease of the saturation drain current was observed. The decay of the current was higher as the dipole moment of the analyte was increasing. Moreover, the morphological surface analysis showed clear differences between PBTTT-C16 and P3HT films that could explain their electrical and sensing behavior. The former exhibited higher responses towards the selected analytes compared to P3HT. This behavior was correlated to the larger voids between the crystal domains of the PBTTT-C16 films and possibly to its higher chemical affinity to the three VOCs. Above all, an enhancement in the response of the sensor was seen in TCBG structures compared to BCBG. The improved sensitivity in this case was attributed either to the contribution of the bulk conductivity and/or to the adsorption of vapor analytes on the gold electrodes.

## Figures and Tables

**Table 1.** Summarized electrical figures of merit for all OTFT devices as calculated from the I_DS_-V_DS_ output characteristics.

Organic Semiconductor		μ (cm^2^/Vs)	V*_T_* (V)	*I*_on_/*I*_off_
PBTTT-C16	TCBG	(7.85 ± 0.02) × 10^−3^	−13.98 ± 0.86	∼ 10^4^
	BCBG	(1.59 ± 0.03) × 10^−2^	−6.69 ± 1.48	∼ 10^4^
P3HT	TCBG	(5.05 ± 0.02) × 10^−4^	−3.52 ± 1.68	∼ 10^3^
	BCBG	(3.81 ± 0.07) × 10^−4^	−2.71 ± 2.01	∼ 10^3^
